# Supplementary material for: Association between pertussis vaccination in infancy and childhood asthma: A population-based record linkage cohort study
Source: PLoS One. 2023 Oct 4;18(10):e0291483. doi: 10.1371/journal.pone.0291483 (PMC10550153; doi:10.1371/journal.pone.0291483)
Supplement: S1 Table — (PDF) [file pone.0291483.s002.pdf]

S1 Table: Eligibility criteria

	Eligibility Criteria
**Inclusion criteria**	1. Birth in WA or NSW between Jan 1, 1997, and Dec 31, 1999<br>2. Receipt of a 1st dose of wP or aP < 4 months old (i.e., < 112 days old), irrespective of subsequent doses
**Exclusion criteria**	1. Born before Jan 1, 1997, or after Dec 31, 1999<br>2. First DTP (wP or aP) < 39 days old<br>3. First DTP (wP or aP) > 112 days old<br>4. First DTP not recorded on AIR

WA: Western Australia. NSW: New South Wales. wP: whole-cell pertussis vaccine. aP: acellular pertussis vaccine. DTP: diphtheria-tetanus-pertussis vaccine. AIR: Australian Immunisation Register.
