# Supplementary material for: Association between pertussis vaccination in infancy and childhood asthma: A population-based record linkage cohort study
Source: PLoS One. 2023 Oct 4;18(10):e0291483. doi: 10.1371/journal.pone.0291483 (PMC10550153; doi:10.1371/journal.pone.0291483)
Supplement: S2 Table — (PDF) [file pone.0291483.s003.pdf]

S2 Table: Study outcomes

Coding scheme	Asthma-related codes
ICD-10 AM	J45, J45.0, J45.1, J45.9, J45.8, J46
SNOMED-CT	304527002, 708038006,99031000119107, 1751000119100, 708093000, 708094006, 135181000119109, 135171000119106, 782513000, 708090002, 10674711000119105, 708095007, 708096008, 10675911000119109, 10675991000119100, 707981009, 10676431000119103, 707980005, 10676511000119109, 10675471000119109, 707979007, 10675551000119104, 733858005, 389145006, 703954005, 703953004, 63088003, 30352005, 195967001, 401000119107, 55570000, 225057002, 233678006, 10692721000119102, 409663006, 762521001, 782520007, 281239006, 425969006, 707445000, 707446004, 707447008, 31387002, 233683003, 424643009, 16055311000119107, 427603009, 125021000119107, 1741000119102, 12428000, 404808000, 233679003, 734904007, 370218001, 427679007, 10675871000119106, 426979002, 10676111000119102, 125011000119100, 10676231000119102, 195977004, 73490500, 370219009, 10676391000119108, 427295004, 125001000119103, 10676671000119102, 10676591000119100, 266361008, 423889005, 16584951000119101, 5281000124103, 901000119100, 445427006, 10675391000119101, 10675431000119106, 426656000, 10675631000119109, 124991000119109, 10675711000119106, 10675751000119107, 2360001000004109, 424199006, 735588005, 707444001, 707511009, 707512002, 735589002, 707513007

Abbreviations: ICD-10 AM, International Classification of Diseases, 10th edition, Australian Modification; SNOMED-CT, Systematized Nomenclature of Medicine, Clinical Terms
