# Supplementary material for: Association between pertussis vaccination in infancy and childhood asthma: A population-based record linkage cohort study
Source: PLoS One. 2023 Oct 4;18(10):e0291483. doi: 10.1371/journal.pone.0291483 (PMC10550153; doi:10.1371/journal.pone.0291483)
Supplement: S3 Table — (PDF) [file pone.0291483.s004.pdf]

S3 Table: Negative control outcomes

Coding scheme	Injury, trauma, or poisoning-related codes
ICD-10 AM	S00 to T98 excluding T78.0, T78.2, T78.3, and T78.4

Abbreviations: ICD-10 AM, International Classification of Diseases, 10th edition, Australian Modification; SNOMED-CT, Systematized Nomenclature of Medicine, Clinical Terms
