# Supplementary material for: Association between pertussis vaccination in infancy and childhood asthma: A population-based record linkage cohort study
Source: PLoS One. 2023 Oct 4;18(10):e0291483. doi: 10.1371/journal.pone.0291483 (PMC10550153; doi:10.1371/journal.pone.0291483)
Supplement: S4 Table — (PDF) [file pone.0291483.s005.pdf]

S4 Table Variable dictionary - Directed acyclic graph

Variable ID	Corresponding node in the DAG	States	Parent nodes
Aboriginal status[a]	Aboriginal status *(L2)*	Aboriginal and/or Torres Strait Islander or non-Aboriginal (reference)	None
Genetic predisposition /Parental asthma[b]	Genetic predisposition *(L5)*	NA	None
State of birth[a]	State of birth *(L21)*	WA or NSW	None
Socio-Economic Indexes for Areas (SEIFA)[a]	Socioeconomic status *(L1)*	0 to 10% (most disadvantaged), 11 to 25%, 26 to 75% (reference), or 91 to 100% (least disadvantaged)	None
Year of birth[a]	Calendar time *(L20)*	1997 (reference), 1998, or 1999	None
Number of previous pregnancies greater than 20 weeks gestation[a]	Birth order *(L4)*	0 (reference), 1, 2, or ≥ 3	None
Season of birth[a]	Season of birth *(L22)*	Summer, autumn, winter (reference), or spring	None
Accessibility/ Remoteness Index of Australia[a]	Remoteness *(L3)*	Major cities (reference), inner and outer regional, or remote and very remote	None
Pertussis vaccine (first dose)[a]	Pertussis vaccine *(A7)*	wP or aP (reference)	Socioeconomic status *(L1)*, calendar time *(L20)*, birth order *(L4)*, state of birth *(L22)*, season of birth *(L21)*, remoteness *(L3)*
Environmental factors	Environmental factors *(L11)*	Nodes: maternal smoking in pregnancy[a], maternal stress[b], viral LRTI[b]	Remoteness *(L3)*, socioeconomic status *(L1)*, genetic predisposition *(L5)*, season of birth *(L22)*
T cell polarization[b]	T cell polarization *(L10)*	NA	Pertussis vaccine *(A7)*, genetic predisposition *(L5)*

S4 Table Variable dictionary - Directed acyclic graph

Variable ID	Corresponding node in the DAG	States	Parent nodes
Asthma[b]	Asthma *(L12)*	NA	T cell polarization *(L10)*, season of birth *(L22)*, environmental factors *(L11)*, birth order *(L4)*, genetic predisposition *(L5)*, socioeconomic status *(L1)*
Access/quality of primary care[b]	Access/quality to primary care *(L8)*	NA	Remoteness *(L3)*, socioeconomic status *(L1)*
Prescription of inhaled corticosteroids (ICS)[b]	Prescription of ICS *(L14)*	NA	Health seeking behavior *(L9)* and access/quality of primary care *(L8)*
Health seeking behavior[b]	Health seeking behavior *(L9)*	NA	Remoteness *(L3)*, birth order *(L4)*, socioeconomic status *(L1)*
Severity of injury[b]	Risk/severity of injury *(L20)*	NA	Remoteness *(L3)*, birth order *(L4)*, socioeconomic status *(L1)*, and Aboriginal status *(L2)*
Treatment adherence[b]	Treatment adherence *(L13)*	NA	Access/quality of primary care *(L8)* and prescription of ICS *(L14)*
Acute asthma exacerbation/ severity[b]	Acute asthma exacerbation/ severity *(L17)*	NA	Environmental factors *(L11)*, asthma *(L12)*, and treatment adherence *(L13)*
Admission to hospital for asthma[b]	Admission to hospital for asthma *(Y18)*	NA	Acute asthma exacerbation/ severity *(L17),* health seeking behavior *(L9),* and threshold for hospitalization *(L16)*
Training for coders[b]	Training for coders *(a15)*	NA	State of birth *(L21)*
Threshold for hospitalization[b]	Threshold for hospitalization *(L16)*	NA	Training for coders *(L15)*, remoteness *(L3)*, socioeconomic status *(L1)*
Admission to hospital ICD-coded as "asthma"[a]	Recorded "asthma" as primary diagnosis in hospital *(Y19)*	Yes or no (reference)	Training for coders *(L15)* and admission to hospital for asthma *(Y18)*
Admissions to hospital ICD-coded as	Recorded "injury, trauma, or poisoning"	Yes or no (reference)	Risk/severity of injury *(L20)*, health seeking behavior *(L9)*,

S4 Table Variable dictionary - Directed acyclic graph

Variable ID	Corresponding node in the DAG	States	Parent nodes
"injury, trauma, or poisoning"[a]	as primary diagnosis in hospital *(Y6)*		threshold for hospitalization *(L16)*, and access/quality of primary care *(L8)*

Abbreviations: ID, identification; DAG, directed acyclic graph; WA, Western Australia; NSW, New South Wales; wP, whole-cell pertussis vaccine; aP, acellular pertussis vaccine; ICS, inhaled corticosteroids; NA, not applicable.

[a]Variable included in the analyzed datasets. [b]Latent variable/mechanism.
