# Supplementary material for: Association between pertussis vaccination in infancy and childhood asthma: A population-based record linkage cohort study
Source: PLoS One. 2023 Oct 4;18(10):e0291483. doi: 10.1371/journal.pone.0291483 (PMC10550153; doi:10.1371/journal.pone.0291483)
Supplement: S5 Table — (PDF) [file pone.0291483.s006.pdf]

S5 Table: Operational definitions

Terms	Operational definition
Confounder	A common cause of the exposure and outcome of interest
Residual confounding	Persistent confounding after adjustment for known and measured confounders
Time-to-first event	The time between cohort entry and the event of interest
Recurrent event	Event that occurs more than once; in this study, recurrent events were defined as those occurring at least 14 days after the previous episode.
