# Supplementary material for: Association between pertussis vaccination in infancy and childhood asthma: A population-based record linkage cohort study
Source: PLoS One. 2023 Oct 4;18(10):e0291483. doi: 10.1371/journal.pone.0291483 (PMC10550153; doi:10.1371/journal.pone.0291483)
Supplement: S6 Table — (PDF) [file pone.0291483.s007.pdf]

S6 Table: Recurrent hospitalizations for asthma among children who received their first pertussis-containing vaccine dose before 4 months old

Number of hospitalizations per child	Study population (N)	Total number of hospitalizations	Complete-case analysis population (N)	Total number of hospitalizations with complete cases
**Overall cohort**				
0	279,367	0	270,450	0
1	3,042	3,042	2,951	2,951
2	638	1,276	624	1,248
≥ 3	393	1,784	380	1,706
**Children vaccinated with wP as a first dose**				
0	189,869	0	183,293	0
1	2,072	2,072	2,007	2,007
2	431	862	421	842
≥ 3	272	1,285	260	1,210
**Children vaccinated with aP as a first dose**				
0	89,498	0	87,157	0
1	970	970	944	944
2	207	414	203	406
≥ 3	121	499	120	496
**Children born in NSW**				
0	214,919	0	212,076	0
1	2,371	2,371	2,354	2,354
2	495	990	493	986
≥ 3	308	1,404	305	1,371
**Children born in WA**				
0	64,448	0	58,374	0
1	671	671	597	597
2	143	286	131	262
≥ 3	85	380	75	335

S6 Table: Recurrent hospitalizations for asthma among children who received their first pertussis-containing vaccine dose before 4 months old

Number of hospitalizations per child	Study population (N)	Total number of hospitalizations	Complete-case analysis population (N)	Total number of hospitalizations with complete cases

Abbreviations: wP, whole-cell pertussis vaccine; aP, acellular pertussis vaccine; NSW: New South Wales; WA, Western Australia
