# Supplementary material for: Association between pertussis vaccination in infancy and childhood asthma: A population-based record linkage cohort study
Source: PLoS One. 2023 Oct 4;18(10):e0291483. doi: 10.1371/journal.pone.0291483 (PMC10550153; doi:10.1371/journal.pone.0291483)
Supplement: S7 Table — (PDF) [file pone.0291483.s008.pdf]

S7 Table: Recurrent hospitalizations for asthma among children vaccinated with a three-dose primary pertussis vaccination series (i.e., any dose of wP versus aP-only doses) before cohort entry (i.e., 5 years old)

Number of hospitalizations per child	Study population (N)	Total number of hospitalizations	Complete-case analysis population (N)	Total number of hospitalizations with complete cases
**Overall cohort**				
0	265,173	0	256,703	0
1	2,873	2,873	2,785	2,785
2	602	1,204	588	1,176
≥ 3	363	1,644	350	1,566
**Children vaccinated with any dose of wP**				
0	193,612	0	186,779	0
1	2,093	2,093	2,019	2,019
2	435	870	424	848
≥ 3	269	1,267	257	1,192
**Children vaccinated with three primary doses of aP**				
0	71,561	0	69,924	0
1	780	780	766	766
2	167	334	164	328
≥ 3	94	377	93	374
**Children born in NSW**				
0	203,877	0	201,181	0
1	2,234	2,234	2,217	2,217
2	468	936	466	932
≥ 3	288	1,306	285	1,273
**Children born in WA**				
0	61,296	0	55,522	0
1	639	639	568	568
2	134	268	122	244
≥ 3	75	338	65	293

S7 Table: Recurrent hospitalizations for asthma among children vaccinated with a three-dose primary pertussis vaccination series (i.e., any dose of wP versus aP-only doses) before cohort entry (i.e., 5 years old)

Number of hospitalizations per child	Study population (N)	Total number of hospitalizations	Complete-case analysis population (N)	Total number of hospitalizations with complete cases

Abbreviations: wP, whole-cell pertussis vaccine; aP, acellular pertussis vaccine; NSW: New South Wales; WA, Western Australia
