# Supplementary material for: Association between pertussis vaccination in infancy and childhood asthma: A population-based record linkage cohort study
Source: PLoS One. 2023 Oct 4;18(10):e0291483. doi: 10.1371/journal.pone.0291483 (PMC10550153; doi:10.1371/journal.pone.0291483)
Supplement: S8 Table — (PDF) [file pone.0291483.s009.pdf]

S8 Table: Recurrent hospitalizations for asthma among children vaccinated with a three-dose primary pertussis vaccination series (i.e., wP-only doses versus aP-only doses) before cohort entry (i.e., 5 years old)

Number of hospitalizations per child	Study population (N)	Total number of hospitalizations	Complete-case analysis population (N)	Total number of hospitalizations with complete cases
**Overall cohort**				
0	204,156	0	197,507	0
1	2,189	2,189	2,127	2,127
2	474	948	463	926
≥ 3	271	1,189	261	1,127
**Children vaccinated with three primary doses of wP**				
0	132,595	0	127,583	0
1	1,409	1,409	1,361	1,361
2	307	614	299	598
≥ 3	177	812	168	753
**Children vaccinated with three primary doses of aP**				
0	71,561	0	69,924	0
1	780	780	766	766
2	167	334	164	328
≥ 3	94	377	93	374
**Children born in NSW**				
0	158,141	0	155,927	0
1	1,711	1,711	1,699	1,699
2	375	750	375	750
≥ 3	222	979	220	951
**Children born in WA**				
0	46,015	0	41,580	0
1	478	478	428	428
2	99	198	88	176
≥ 3	49	210	41	176

S8 Table: Recurrent hospitalizations for asthma among children vaccinated with a three-dose primary pertussis vaccination series (i.e., wP-only doses versus aP-only doses) before cohort entry (i.e., 5 years old)

Number of hospitalizations per child	Study population (N)	Total number of hospitalizations	Complete-case analysis population (N)	Total number of hospitalizations with complete cases

Abbreviations: wP, whole-cell pertussis vaccine; aP, acellular pertussis vaccine; NSW: New South Wales; WA, Western Australia
