# Supplementary material for: Association between pertussis vaccination in infancy and childhood asthma: A population-based record linkage cohort study
Source: PLoS One. 2023 Oct 4;18(10):e0291483. doi: 10.1371/journal.pone.0291483 (PMC10550153; doi:10.1371/journal.pone.0291483)
Supplement: S9 Table — (PDF) [file pone.0291483.s010.pdf]

S9 Table: NSW cohort - Recurrent presentations to the emergency department for asthma among children who received their first pertussis-containing vaccine dose before 4 months old

Number of presentations per child	Study population (N)	Total number of presentations	Complete-case analysis population (N)	Total number of presentations with complete cases (n)
**Overall cohort**				
0	214,394	0	211,556	0
1	2,690	2,690	2,674	2,674
2	551	1,102	547	1,094
≥ 3	399	1,741	394	1,705
**Children vaccinated with wP as a first dose**				
0	145,881	0	143,877	0
1	1,841	1,841	1,828	1,828
2	374	748	370	740
≥ 3	278	1,293	274	1,260
**Children vaccinated with aP as a first dose**				
0	68,513	0	67,679	0
1	849	849	846	846
2	177	354	177	354
≥ 3	121	448	120	445

Abbreviations: wP, whole-cell pertussis vaccine; aP, acellular pertussis vaccine
