# Supplementary material for: Association between pertussis vaccination in infancy and childhood asthma: A population-based record linkage cohort study
Source: PLoS One. 2023 Oct 4;18(10):e0291483. doi: 10.1371/journal.pone.0291483 (PMC10550153; doi:10.1371/journal.pone.0291483)
Supplement: S10 Table — (PDF) [file pone.0291483.s011.pdf]

S10 Table: NSW cohort - Recurrent presentations to the emergency department for asthma among children receiving a three-dose primary pertussis vaccination series (i.e., any dose of wP versus aP-only doses) before cohort entry (i.e., 8 years old)

Number of presentations per child	Study population (N)	**Total number of presentations**	**Complete-case analysis population (N)**	**Total number of presentations with complete cases (n)**
**Overall cohort**				
0	203,414	0	200,723	0
1	2,550	2,550	2,534	2,534
2	524	1,048	520	1,040
≥ 3	367	1,595	362	1,559
**Children vaccinated with any dose of wP**				
0	147,127	0	145,148	0
1	1,851	1,851	1,838	1,838
2	374	748	370	740
≥ 3	270	1,239	266	1,206
**Children vaccinated with three primary doses of aP**				
0	56,287	0	55,575	0
1	699	699	696	696
2	150	300	150	300
≥ 3	97	356	96	353

Abbreviations: wP, whole-cell pertussis vaccine; aP, acellular pertussis vaccine
