# Supplementary material for: Association between pertussis vaccination in infancy and childhood asthma: A population-based record linkage cohort study
Source: PLoS One. 2023 Oct 4;18(10):e0291483. doi: 10.1371/journal.pone.0291483 (PMC10550153; doi:10.1371/journal.pone.0291483)
Supplement: S11 Table — (PDF) [file pone.0291483.s012.pdf]

S11 Table: NSW cohort - Recurrent presentations to the emergency department for asthma among children receiving a three-dose primary pertussis vaccination series (i.e., wP-only doses versus aP-only doses) before cohort entry (i.e., 8 years old)

Number of presentations per child	Study population (N)	Total number of presentations	Complete-case analysis population (N)	Total number of presentations with complete cases (n)
**Overall cohort**				
0	157,770	0	155,561	0
1	1,951	1,951	1,937	1,937
2	422	844	420	840
≥ 3	274	1,178	272	1,155
**Children vaccinated with three primary doses of wP**				
0	101,483	0	99,986	0
1	1,252	1,252	1,241	1,241
2	272	544	270	540
≥ 3	177	822	176	802
**Children vaccinated with three primary doses of aP**				
0	56,287	0	55,575	0
1	699	699	696	696
2	150	300	150	300
≥ 3	97	356	96	353

Abbreviations: wP, whole-cell pertussis vaccine; aP, acellular pertussis vaccine
