# Supplementary material for: Association between pertussis vaccination in infancy and childhood asthma: A population-based record linkage cohort study
Source: PLoS One. 2023 Oct 4;18(10):e0291483. doi: 10.1371/journal.pone.0291483 (PMC10550153; doi:10.1371/journal.pone.0291483)
Supplement: S12 Table — (PDF) [file pone.0291483.s013.pdf]

**S12 Table: WA cohort - Recurrent presentations to the emergency department for asthma among children receiving at least one dose of pertussis-containing vaccine before 4 months old**

| Number of presentations per child | Study population (N) | Total number of presentations | Complete-case analysis population (N) | Total number of presentations with complete cases (n) |
|---|---|---|---|---|
| **Overall cohort** | | | | |
| 0 | 64,607 | 0 | 58,484 | 0 |
| 1 | 568 | 568 | 533 | 533 |
| 2 | 105 | 210 | 99 | 198 |
| ≥ 3 | 67 | 284 | 61 | 263 |
| **Children vaccinated with wP as a first dose** | | | | |
| 0 | 43,731 | 0 | 39,127 | 0 |
| 1 | 372 | 372 | 348 | 348 |
| 2 | 81 | 162 | 77 | 154 |
| ≥ 3 | 50 | 219 | 45 | 201 |
| **Children vaccinated with aP as a first dose** | | | | |
| 0 | 20,876 | 0 | 19,357 | 0 |
| 1 | 196 | 196 | 185 | 185 |
| 2 | 24 | 48 | 22 | 44 |
| ≥ 3 | 17 | 65 | 16 | 62 |

Abbreviations: wP, whole-cell pertussis vaccine; aP, acellular pertussis vaccine
