# Supplementary material for: Association between pertussis vaccination in infancy and childhood asthma: A population-based record linkage cohort study
Source: PLoS One. 2023 Oct 4;18(10):e0291483. doi: 10.1371/journal.pone.0291483 (PMC10550153; doi:10.1371/journal.pone.0291483)
Supplement: S13 Table — (PDF) [file pone.0291483.s014.pdf]

S13 Table: WA cohort - Recurrent presentations to the emergency department for asthma among children receiving three-dose primary pertussis vaccination series (i.e., any dose of wP versus aP-only doses) before cohort entry (i.e., 5 years old)

Number of presentations per child	Study population (N)	Total number of presentations	Complete-case analysis population (N)	Total number of presentations with complete cases (n)
**Overall cohort**				
0	61,442	0	55,621	0
1	539	539	504	504
2	100	200	94	188
≥ 3	63	264	58	248
**Children vaccinated with any dose of wP**				
0	46,263	0	41,369	0
1	389	389	362	362
2	80	160	76	152
≥ 3	53	223	49	210
**Children vaccinated with three primary doses of aP**				
0	15,179	0	14,252	0
1	150	150	142	142
2	20	40	18	36
≥ 3	10	41	9	38

Abbreviations: wP, whole-cell pertussis vaccine; aP, acellular pertussis vaccine
