# Supplementary material for: Association between pertussis vaccination in infancy and childhood asthma: A population-based record linkage cohort study
Source: PLoS One. 2023 Oct 4;18(10):e0291483. doi: 10.1371/journal.pone.0291483 (PMC10550153; doi:10.1371/journal.pone.0291483)
Supplement: S14 Table — (PDF) [file pone.0291483.s015.pdf]

S14 Table: WA cohort - Recurrent presentations to the emergency department for asthma among children receiving a three-dose primary pertussis vaccination series (i.e., wP-only doses versus aP-only doses) before cohort entry (i.e., 5 years old)

Number of presentations per child	Study population (N)	Total number of presentations	Complete-case analysis population (N)	Total number of presentations with complete cases (n)
**Overall cohort**				
0	46,117	0	41,649	0
1	413	413	385	385
2	71	142	66	132
≥ 3	40	164	37	154
**Children vaccinated with three primary doses of wP**				
0	30,938	0	27,397	0
1	263	263	243	243
2	51	102	48	96
≥ 3	30	123	28	116
**Children vaccinated with three primary doses of aP**				
0	15,179	0	14,252	0
1	150	150	142	142
2	20	40	18	36
≥ 3	10	41	9	38

Abbreviations: wP, whole-cell pertussis vaccine; aP, acellular pertussis vaccine
