# Supplementary material for: Association between pertussis vaccination in infancy and childhood asthma: A population-based record linkage cohort study
Source: PLoS One. 2023 Oct 4;18(10):e0291483. doi: 10.1371/journal.pone.0291483 (PMC10550153; doi:10.1371/journal.pone.0291483)
Supplement: S15 Table — (PDF) [file pone.0291483.s016.pdf]

S15 Table: NSW and WA cohorts - Hazard ratios and 95% confidence intervals for emergency department presentations for asthma (cohort entry: 8 years old)

Exposure - Outcome	Analysis population (N)[a]	Presentations (n)	Incidence rate (95% CI) per 1,000 child-years	Unadjusted HR (95% CI)[b]	Adjusted HR (95% CI)[c]
**wP versus aP as a first dose - Time-to-first presentation**					
aP	88,396	1,227	2.1 (2–2.2)	1 [Reference]	1 [Reference]
wP	185,933	2,693	2.1 (2–2.2)	1.01 (0.94–1.08)	0.96 (0.88–1.08)
**wP versus aP as a first dose - Time-to-recurrent presentations**					
aP	88,396	1,750	3 (2.9–3.2)	1 [Reference]	1 [Reference]
wP	185,933	4,129	3.2 (3.1–3.3)	1.08 (1–1.18)	1.05 (0.93–1.17)
**Any wP versus all aP - Time-to-first presentation**					
aP/aP/aP	70,937	999	2.2 (2–2.3)	1 [Reference]	1 [Reference]
Any wP	189,480	2,703	2.1 (2–2.2)	0.98 (0.91–1.05)	0.97 (0.88–1.07)
**Any wP versus all aP - Time-to-recurrent presentations**					
aP/aP/aP	70,937	1,417	3.1 (2.9–3.2)	1 [Reference]	1 [Reference]
Any wP	189,480	4,096	3.1 (3–3.2)	1.04 (0.95–1.14)	1.05 (0.93–1.18)
**All wP versus all aP - Time-to-first presentation**					
aP/aP/aP	70,937	999	2.2 (2–2.3)	1 [Reference]	1 [Reference]
wP/wP/wP	129,380	1,834	2.1 (2–2.1)	0.96 (0.89–1.04)	0.94 (0.82–1.07)
**All wP versus all aP - Time-to-recurrent presentations**					
aP/aP/aP	70,937	1,417	3.1 (2.9–3.2)	1 [Reference]	1 [Reference]
wP/wP/wP	129,380	2,776	3.1 (3–3.2)	1.02 (0.93–1.13)	1.05 (0.89–1.23)

Abbreviations: CI, confidence interval; HR, hazard ratio; wP, whole-cell pertussis vaccine; aP, acellular pertussis vaccine.

[a]The analysis population included only those without missing data.

[b]Unadjusted HRs were calculated with all the eligible members of the cohort.

S15 Table: NSW and WA cohorts - Hazard ratios and 95% confidence intervals for emergency department presentations for asthma (cohort entry: 8 years old)

Exposure - Outcome	Analysis population (N)[a]	Presentations (n)	Incidence rate (95% CI) per 1,000 child-years	Unadjusted HR (95% CI)[b]	Adjusted HR (95% CI)[c]

[c]Adjusted HRs were calculated with complete cases. The multivariable models were adjusted for year of birth, birth order (using number of previous pregnancies as a surrogate), maternal smoking during pregnancy, socioeconomic status, the index of accessibility/remoteness of Australia, sex, Aboriginal status, delivery method, season of birth, and gestational age as a penalized spline.
