# Supplementary material for: Association between pertussis vaccination in infancy and childhood asthma: A population-based record linkage cohort study
Source: PLoS One. 2023 Oct 4;18(10):e0291483. doi: 10.1371/journal.pone.0291483 (PMC10550153; doi:10.1371/journal.pone.0291483)
Supplement: S16 Table — (PDF) [file pone.0291483.s017.pdf]

S16 Table: Hazard ratios and 95% confidence intervals for hospitalizations for injury, trauma, or poisoning

Exposure - Outcome	Analysis population (N)[a]	Hospitalizations (n)	Incidence rate (95% CI) per 1,000 child-years	Unadjusted HR (95% CI)[b]	Adjusted HR (95% CI)[c]
**wP versus aP as a first dose - Time-to-first admission**					
aP	88,424	8,544	10.6 (10.4–10.8)	1 [Reference]	1 [Reference]
wP	185,981	19,191	10.9 (10.8–11.1)	1 (0.94–1.07)	0.97 (0.94–1.01)
**wP versus aP as a first dose - Time-to-recurrent presentation**					
aP	88,424	9,708	11.5 (11.2–11.7)	1 [Reference]	1 [Reference]
wP	185,981	21,961	11.9 (11.7–12)	1.02 (0.99–1.04)	0.97 (0.93–1)
**Any wP versus all aP - Time-to-first admission**					
aP/aP/aP	70,947	6,781	10.5 (10.3–10.8)	1 [Reference]	1 [Reference]
Any wP	189,479	19,575	11 (10.8–11.1)	1.04 (1.01–1.06)	0.99 (0.95–1.03)
**Any wP versus all aP - Time-to-recurrent presentation**					
aP/aP/aP	70,947	7,700	11 (11–12)	1 [Reference]	1 [Reference]
Any wP	189,479	22,384	12 (12–12)	1.03 (1.00-1.06)	0.98 (0.95–1.02)
**All wP versus all aP - Time-to-first admission**					
wP/wP/wP	70,947	6,781	10.5 (10.3–10.8)	1 [Reference]	1 [Reference]
aP/aP/aP	129,411	13,306	10.9 (106.8–11.1)	1.03 (1–1.06)	0.95 (0.91–1)
**All wP versus all aP - Time-to-recurrent presentation**					
wP/wP/wP	70,947	7,700	11.4 (11.1–11.6)	1 [Reference]	1 [Reference]
aP/aP/aP	129,411	15,245	11.8 (11.6–12)	1.02 (0.99-1.05)	0.94 (0.9-0.99)

S16 Table: Hazard ratios and 95% confidence intervals for hospitalizations for injury, trauma, or poisoning

Exposure - Outcome	Analysis population (N)[a]	Hospitalizations (n)	Incidence rate (95% CI) per 1,000 child-years	Unadjusted HR (95% CI)[b]	Adjusted HR (95% CI)[c]

Abbreviations: CI, confidence interval; HR, hazard ratio; wP, whole-cell pertussis vaccine; aP, acellular pertussis vaccine.

[a]The analysis population included only those without missing data.

[b]Unadjusted HRs were calculated with all the eligible members of the cohort.

[c]Adjusted HRs were calculated with complete cases. The multivariable models were adjusted for year of birth, birth order (using number of previous pregnancies as a surrogate), maternal smoking during pregnancy, socioeconomic status, the index of accessibility/remoteness of Australia, sex, Aboriginal status, delivery method, season of birth, and gestational age as a penalized spline.
