# Supplementary figures and images for: Association between pertussis vaccination in infancy and childhood asthma: A population-based record linkage cohort study
Source: PLoS One. 2023 Oct 4;18(10):e0291483. doi: 10.1371/journal.pone.0291483 (PMC10550153; doi:10.1371/journal.pone.0291483)

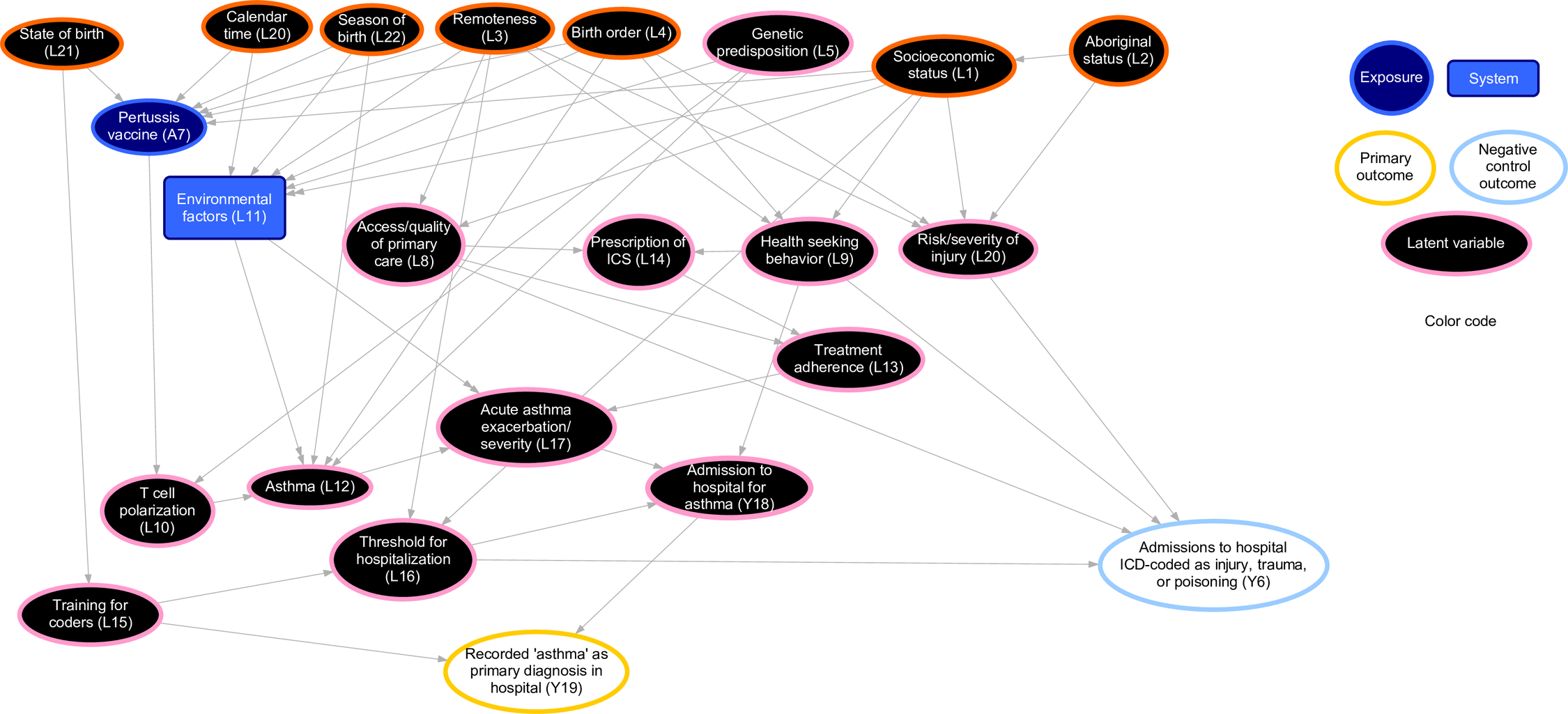

Supplement: S1 Fig — Simplified directed acyclic graph describing the proposed causal relationship between pertussis immunization and admissions to hospital with an assigned principal diagnosis of asthma according to the International Classification of Diseases (9th edition, Clinical Modification or 10th edition, Australian Modification), in children born in Western Australia (L21 = 0) or New South Wales (L21 = 1) between 1997 and 1999 (L20). Admissions to hospital with an assigned principal diagnosis of injury, trauma, or poisoning per the same coding schemes represent the negative control outcome of this study (Y6). These outcomes are not in the causal pathway between the exposure of interest (A7) and admissions for asthma (Y18 or its descendant Y19), despite sharing a set of common causes. Figure generated via GeNIe Academic version 4.0.1922.0.[1]. 1BayesFusion. GeNIe Modeler [Internet]. 2022. Available from: https://www.bayesfusion.com/genie/ . (TIF) [file pone.0291483.s018.tif]

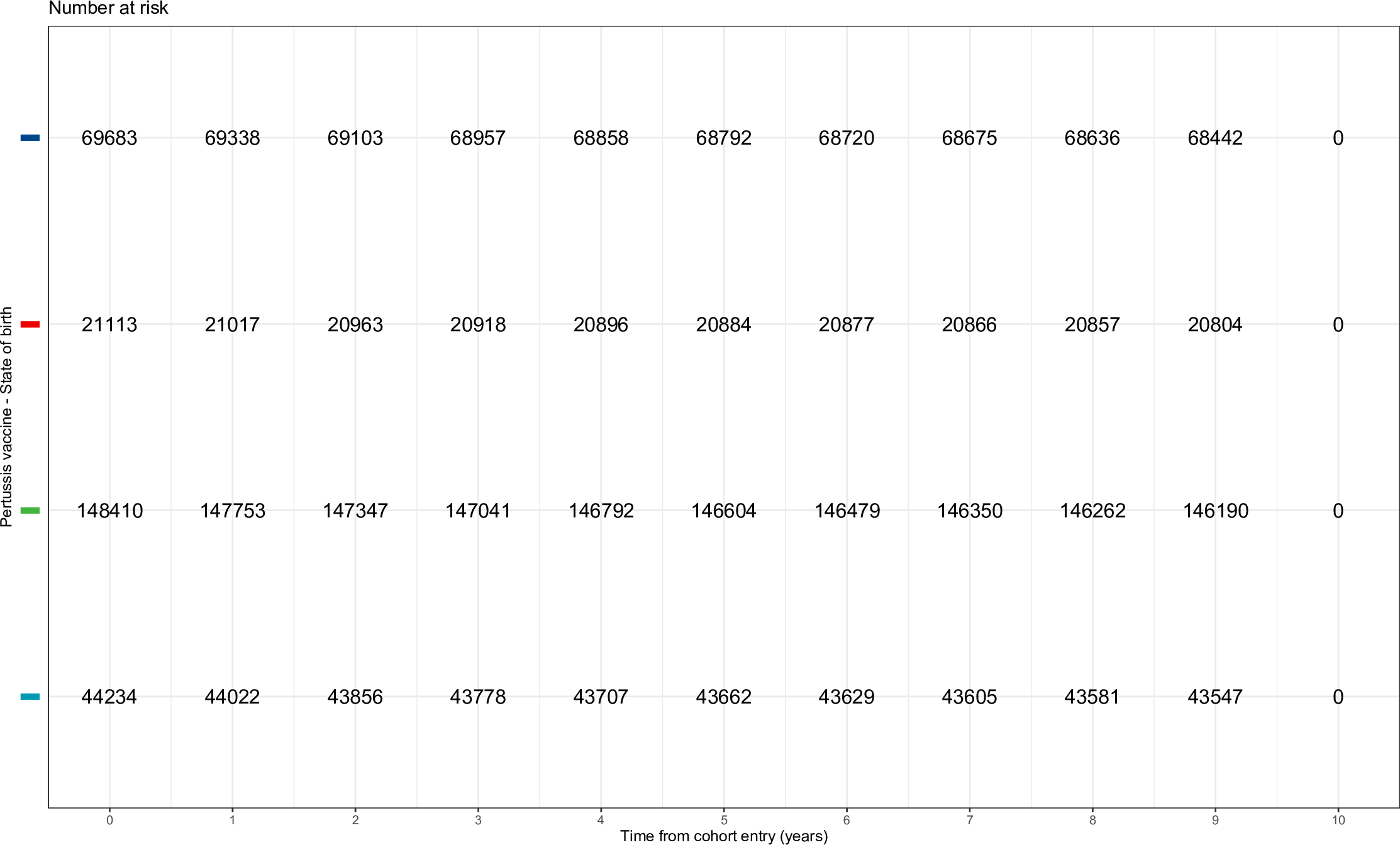

Supplement: S2 Fig — (A) Time-to-first hospitalization for asthma among children born in New South Wales or Western Australia between 1997 and 1999 stratified by state of birth. wP as a first dose versus aP as a first dose. wP: whole-cell pertussis vaccine. aP: acellular pertussis vaccine. (B) Number at risk table. This figure was generated in R using the tidyverse core packages, as well as the survival and survminer libraries [1–4]. 1R Core Team. R: A Language and Environment for Statistical Computing [Internet]. Vienna, Austria: R Foundation for Statistical Computing; 2022. Available from: https://www.R-project.org/. 2Wickham H, Averick M, Bryan J, Chang W, D’Agostino McGowan L, François R, Müller K et al. Welcome to the tidyverse. J. Open Source Softw. 2019; 4(43), 1686. doi:10.21105/joss.01686. 3Therneau T. A package for survival analysis in R [Internet]. R package version 3.5–5. Available from: https://CRAN.R-project.org/package=survival. 4Kassambara A, Kosinski M, Przemyslaw B. survminer: Drawing Survival Curves using ’ggplot2’. R package version 0.4.9. [Internet]. 2021. Available from: https://CRAN.R-project.org/package=survminer . (ZIP) [file pone.0291483.s019.zip › S2_Figure_panel_B.tif]

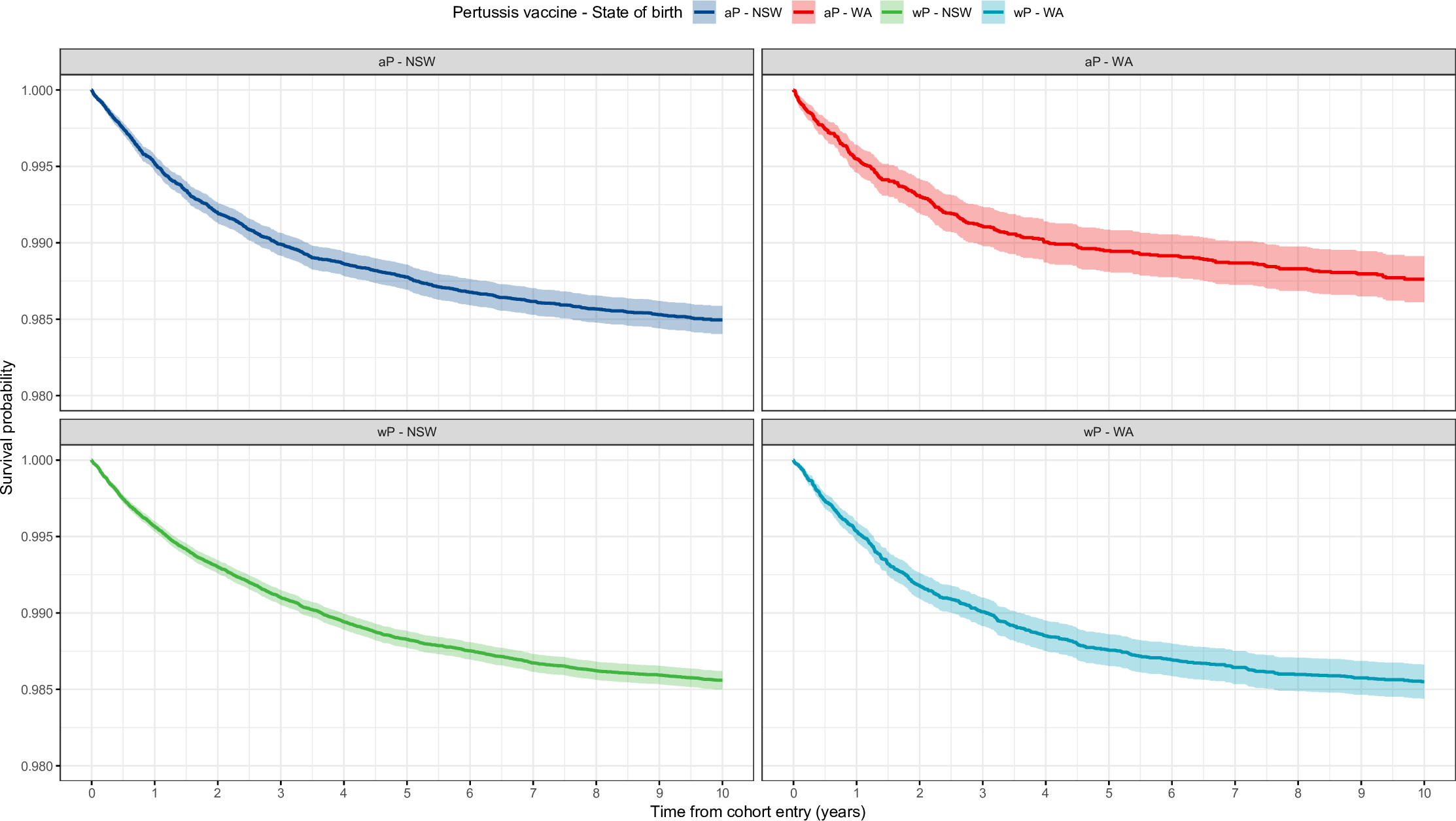

Supplement: S2 Fig — (A) Time-to-first hospitalization for asthma among children born in New South Wales or Western Australia between 1997 and 1999 stratified by state of birth. wP as a first dose versus aP as a first dose. wP: whole-cell pertussis vaccine. aP: acellular pertussis vaccine. (B) Number at risk table. This figure was generated in R using the tidyverse core packages, as well as the survival and survminer libraries [1–4]. 1R Core Team. R: A Language and Environment for Statistical Computing [Internet]. Vienna, Austria: R Foundation for Statistical Computing; 2022. Available from: https://www.R-project.org/. 2Wickham H, Averick M, Bryan J, Chang W, D’Agostino McGowan L, François R, Müller K et al. Welcome to the tidyverse. J. Open Source Softw. 2019; 4(43), 1686. doi:10.21105/joss.01686. 3Therneau T. A package for survival analysis in R [Internet]. R package version 3.5–5. Available from: https://CRAN.R-project.org/package=survival. 4Kassambara A, Kosinski M, Przemyslaw B. survminer: Drawing Survival Curves using ’ggplot2’. R package version 0.4.9. [Internet]. 2021. Available from: https://CRAN.R-project.org/package=survminer . (ZIP) [file pone.0291483.s019.zip › S2_Figure_panel_A.tif]
