# Supplementary material for: Association between pertussis vaccination in infancy and childhood asthma: A population-based record linkage cohort study
Source: PLoS One. 2023 Oct 4;18(10):e0291483. doi: 10.1371/journal.pone.0291483 (PMC10550153; doi:10.1371/journal.pone.0291483)
Supplement: S1 File — (ZIP) [file pone.0291483.s020.zip › S1_Supporting_Information_A.html]

Data visualization


# Data visualization

### Association between pertussis vaccination in infancy and childhood asthma: a population-based record linkage study

```
knitr::opts_chunk$set(echo = TRUE)
library (tidyverse)
```

```
## -- Attaching packages --------------------------------------- tidyverse 1.3.1 --
```

```
## v ggplot2 3.3.5     v purrr   0.3.4
## v tibble  3.1.4     v dplyr   1.0.7
## v tidyr   1.1.3     v stringr 1.4.0
## v readr   2.0.1     v forcats 0.5.1
```

```
## -- Conflicts ------------------------------------------ tidyverse_conflicts() --
## x dplyr::filter() masks stats::filter()
## x dplyr::lag()    masks stats::lag()
```

```
library (survival)
library(survminer)
```

```
## Loading required package: ggpubr
```

```
## 
## Attaching package: 'survminer'
```

```
## The following object is masked from 'package:survival':
## 
##     myeloma
```

```
test1 <-
  readRDS("/Analysis/Glady PhD Analysis/Analysis/Test to discuss/Hospital/Models/Asthma/Model 1/Bivariate/Outputs/01_data_uv_m1.rds") %>%
  mutate(tstop1 = tstop1 / 365.25)

fit <- survfit(Surv(tstop1, new_asthma) ~ dosetype1_f, data = test1)
fit1 <- survfit(Surv(tstop1, new_asthma) ~ dosetype1_f + strata(stateorigin_f), data = test1)
```

```
ggsurvplot(fit,
  title = 'Unadjusted primary analysis',
  subtitle = "Time-to-first admission to hospital ICD-coded as asthma",
  palette = "lancet",
  risk.table = "absolute",
  risk.table.fontsize = 3,
  risk.table.height = 0.3,
  risk.table.y.text.col = TRUE,
  risk.table.y.text = FALSE,
  break.time.by = 1,
  censor = FALSE,
  ylim = c(.98, 1),
  xlab = "Years since cohort entry",
  legend.labs = c("aP (first dose)", "wP (first dose)"),
  legend.title = "Pertussis vaccine",
  conf.int = TRUE,
  ggtheme = theme_bw())
```

```
names(fit1$strata)[[1]] <- "aP - NSW"
names(fit1$strata)[[2]] <- "aP - WA"
names(fit1$strata)[[3]] <- "wP - NSW"
names(fit1$strata)[[4]] <- "wP - WA"


aa = ggsurvplot(fit1,
           title = 'Exploratory analysis stratified by state of birth',
           subtitle = "Time-to-first admission to hospital ICD-coded as asthma",
           palette = "lancet",
           risk.table = "absolute",
           risk.table.fontsize = 3,
           risk.table.height = 0.1,
           risk.table.y.text.col = TRUE,
           risk.table.y.text = FALSE,
           break.time.by = 1,
           censor = FALSE,
           ylim = c(.98, 1),
           xlab = "Years since cohort entry",
           legend.title = "Pertussis vaccine - State of birth",
           conf.int = TRUE,
           ggtheme = theme_bw())


aa$plot + facet_wrap(~strata)
```

```
aa$table
```
