# Supplementary material for: Association between pertussis vaccination in infancy and childhood asthma: A population-based record linkage cohort study
Source: PLoS One. 2023 Oct 4;18(10):e0291483. doi: 10.1371/journal.pone.0291483 (PMC10550153; doi:10.1371/journal.pone.0291483)
Supplement: Attachment — Submitted filename: Reviewer response for plos(3x).docx [file pone.0291483.s021.docx]

**Reviewer response**

Association between pertussis vaccination in infancy and asthma: a population-based record linkage cohort study

**comments**

- I really appreciate the research idea however the topic needs a bit of improvement.

**Introduction section**

It is well written however it is more clinical hence authors need to show the asthma burden among school children, what are the health consequences, and the economic burden of it.

Why did you prefer to conduct ages between 5 to 15 years children?

Your outcome variable is not clearly stated so you need to write clearly again.

I didn’t see the clearly stated inclusion and exclusion criteria so you better follow the guideline of the journal.

I found two outcome variables such as time to 1^st^ event and time to recurrent event so how did you manage it? I think it is somewhat vast. Both might have different factors that you have to analyze, show results, and discuss separately.

There is no independent operational definition section and you fail to operationalize some concepts that you mention in the document for instance residual confounder, first event of time, recurrent event

**Regarding method section**

I didn’t find a data extraction tool with its description. I think it has to be incorporated in this section.

Again, you fail to describe how to control data quality so you have to write it.

In the data analysis and process section: some indispensable points are overlooked or not written like model fitness, what were you assumption to take candidate variables from bivariate to multivariate Cox regression statistical model, VIF, AND proportional hazard assumptions?

**Result section**

How much data you enrolled from that how much data you analyzed?

There are two tables that existed in your document but it doesn’t show the succession which means you put table 1 and table 1 so at least you need to show the continuity of the table by using the word “table 1 continued”.

Since your study is about survival, where are the important graphs that show your study like Kaplan Meir survival estimate, cox regression table, and Comparison of survivorship functions for different categorical variables?

**Discussion section**

It is shallow to reveal the detail of your findings

**References**

Of the total of 3,4,13,14,15,20, and 22 references are outdated which is not appropriate.
