# Supplementary material for: Association between pertussis vaccination in infancy and childhood asthma: A population-based record linkage cohort study
Source: PLoS One. 2023 Oct 4;18(10):e0291483. doi: 10.1371/journal.pone.0291483 (PMC10550153; doi:10.1371/journal.pone.0291483)
Supplement: Attachment — Submitted filename: Response to Reviewers.docx [file pone.0291483.s022.docx]

REVIEWERS’ COMMENTS

Re: PONE-D-23-11079. “Association between pertussis vaccination in infancy and childhood asthma: a population-based record linkage cohort study“.

**To maintain the integrity of the review process, the comments from the reviewers are included verbatim using quotation marks.**

**REVIEWER # 1**

| *Reviewer #1 – Comments from the reviewer are included verbatim* | *Authors’ reply* |
| --- | --- |
| *“Association between pertussis vaccination in infancy and asthma: a population-based record linkage cohort study comments. We really appreciate the research idea however the topic needs a bit of improvement.”* | 1. We thank the reviewer for their time and comments. 2. Where the answers to the reviewer questions were included in the original manuscript, we copy them below to facilitate the review process. 3. Unless otherwise stated, the line numbers listed below refer to the unmarked version of our revised paper. 4. Please note that the number of some citations changed, as we added new references to our manuscript. |
| *“Introduction section”*  *“It is well written however it is more clinical hence authors need to show the asthma burden among school children, what are the health consequences, and the economic burden of it.”* | The following paragraph has been added to the introduction (lines 54 to 64).  *The Australian National Health Survey (2007-2008) estimated that 10.4% (95% confidence interval [CI] 9.1% to 11.7%) of children under 15 years old had current asthma,^3^ a pattern that remained unaltered in the subsequent decade.^4^ In 2015, the Australian health expenditure associated with asthma was $AU770 million, with at least one-third utilised in hospital services.^5^ In the same setting, self-reported asthma was the leading cause of the total burden of disease due to respiratory illness in children aged between 5 and < 15 years old and the leading cause of non-fatal disease burden among those under 15 years old.5 Uncontrolled childhood asthma is associated with sleep disturbances (49.7%; 95% CI 48.8% to 50.6%) and school absenteeism (46.3%; 95% CI 45.4% to 47.1%).^6^ Furthermore, early persistent asthma or persistent wheezing disorders have been associated with impaired lung development throughout childhood and adolescence, further impacting lung function in adulthood.^7^* |
| *“Why did you prefer to conduct ages between 5 to 15 years children?”* | The answer to the reviewer’s question was included in the original manuscript (i.e., Introduction section, lines 70 to 73 in the original manuscript): “We were interested in assessing protection against asthma in school-aged children, because many wheezing episodes in younger children are driven by viral respiratory infections without upregulation of Th2 associated-pathways.^8^ Nonetheless, for readability and clarity we reword it as follows (lines 85 to 88): *“We were interested in assessing protection against asthma in children aged between 5 and < 15 years old, because most wheezing episodes in younger children are driven by viral respiratory infections without upregulation of Th2-associated pathways.^15^” We also edited the title of this manuscript to emphasise the above: “Association between pertussis vaccination in infancy* ***and childhood asthma****: a population-based record linkage cohort study“.* |
| *“Your outcome variable is not clearly stated so you need to write clearly again.”* | The Outcomes section has been edited. It now reads:  Hospitalizations (lines 130 to 133)  *The outcomes of interest were first and recurrent hospitalizations assigned a primary diagnosis of asthma per ICD-10 AM (S2 Table). Recurrent events were defined as those occurring at least 14 days after the previous episode.*  Emergency Department Presentations (lines 137 to 139)  *“First and recurrent presentations to the emergency department for asthma were also examined as study outcomes. Recurrent events were defined as above.”* |
| *“I didn’t see the clearly stated inclusion and exclusion criteria so you better follow the guideline of the journal.”* | Below we include both, the cohort’s eligibility criteria stated in the original submission and the operational definitions requested by the reviewer (lines 101 to 108).  *“The cohort was identified through the birth registries of the Australian states of New South Wales (NSW) and Western Australia (WA), and the NSW Perinatal Data Collection and WA Midwives’ Notification System. The linkage accuracy between birth register and immunization datasets was 99.0%, and between the birth registry and death data 96.6%.^17^ We included all children born between January 1, 1997, and December 31, 1999, who received a first dose of either wP or aP-containing vaccine before 4 months old, irrespective of subsequent doses, and had records in the perinatal and birth registries. Inclusion and exclusion criteria are operationalized in the Supplement (S1 Table).”* |

***S1 Table, Eligibility criteria***

|  | ***Eligibility Criteria*** |
| --- | --- |
| ***Inclusion criteria*** | 1. *Birth in WA or NSW between Jan 1, 1997, and Dec 31, 1999* 2. *Receipt of a 1^st^ dose of wP or aP < 4 months old (i.e., < 112 days old), irrespective of subsequent doses* |
| ***Exclusion criteria*** | 1. *Born before Jan 1, 1997, or after Dec 31, 1999* 2. *First DTP (wP or aP) < 39 days old* 3. *First DTP (wP or aP) > 112 days old* 4. *First DTP not recorded on AIR* |

WA: Western Australia. NSW: New South Wales. wP: whole-cell pertussis vaccine. aP: acellular pertussis vaccine. DTP: diphtheria-tetanus-pertussis vaccine. AIR: Australian Immunisation Register.

| *Reviewer #1 – Comments from the reviewer are included verbatim* | *Authors’ reply* |
| --- | --- |
| *“I found two outcome variables such as time to 1st event and time to recurrent event so how did you manage it? I think it is somewhat vast. Both might have different factors that you have to analyze, show results, and discuss separately.”* | *“How did you manage?”* Lines 139 to 142 from the original manuscript answer the question. *“Time-to-first event and time-to-recurrent events analyses were conducted using Cox and Andersen-Gill regression models, respectively, with robust variance estimates to account for potential within-child dependencies.”* |
| *Both might have different factors that you have to analyze, show results, and discuss separately.”* | 1. The analyses were pre-specified in the protocol. 2. The model assumptions for both, time-to-first event and time-to-recurrent event analyses were made publicly available as DAGs and extensively discussed in the study protocol. 3. Recurrent events and time-to-recurrent-event analyses were all summarised in the original submission. These can be found in:  - Table 2: Hazard ratios and 95% confidence intervals for hospitalizations for asthma - Table 3: Hazard ratios and 95% confidence intervals for emergency department presentations for asthma in children born in New South Wales between 1997 and 1999 - Table 4: Hazard ratios and 95% confidence intervals for emergency department presentations for asthma in children born in Western Australia between 1997 and 1999 - S6 Table: Recurrent hospitalizations for asthma among children who received their first pertussis-containing vaccine dose before 4 months old - S7 Table: Recurrent hospitalizations for asthma among children vaccinated with a three-dose primary pertussis vaccination series (i.e., any dose of wP versus aP-only doses) before cohort entry (i.e., 5 years old) - S8 Table: Recurrent hospitalizations for asthma among children vaccinated with a three-dose primary pertussis vaccination series (i.e., wP-only doses versus aP-only doses) before cohort entry (i.e., 5 years old) - S9 Table: NSW cohort - Recurrent presentations to the emergency department for asthma among children who received their first pertussis-containing vaccine dose before 4 months old - S10 Table: NSW cohort - Recurrent presentations to the emergency department for asthma among children receiving a three-dose primary pertussis vaccination series (i.e., any dose of wP versus aP-only doses) before cohort entry (i.e., 8 years old) - S11 Table: NSW cohort - Recurrent presentations to the emergency department for asthma among children receiving a three-dose primary pertussis vaccination series (i.e., wP-only doses versus aP-only doses) before cohort entry (i.e., 8 years old) - S12 Table: WA cohort - Recurrent presentations to the emergency department for asthma among children receiving at least one dose of pertussis-containing vaccine before 4 months old - S13 Table: WA cohort - Recurrent presentations to the emergency department for asthma among children receiving three-dose primary pertussis vaccination series (i.e., any dose of wP versus aP-only doses) before cohort entry (i.e., 5 years old) - S14 Table: WA cohort - Recurrent presentations to the emergency department for asthma among children receiving a three-dose primary pertussis vaccination series (i.e., wP-only doses versus aP-only doses) before cohort entry (i.e., 5 years old) - S15 Table: NSW and WA cohorts - Hazard ratios and 95% confidence intervals for emergency department presentations for asthma (cohort entry: 8 years old)  4. The results were also discussed separately in the original submission, as shown in the first paragraph of the discussion: “*In this population-based cohort study, we found little evidence of a clinically relevant difference in the risk of hospitalization or emergency department presentation for asthma among wP or aP-vaccinated children.* ***There was evidence of a modestly increased rate of recurrent emergency department presentation for asthma among WA-born children vaccinated with a first dose of wP versus aP, but this was not observed for time-to-first emergency presentation, nor was it observed among the NSW cohort. Considering the totality of the evidence, we doubt this reflects a true difference in risk for wP and aP-vaccinated children born in WA****.”* |
| *“There is no independent operational definition section and you fail to operationalize some concepts that you mention in the document for instance residual confounder, first event of time, recurrent event.”* | *We have now added the following Table (S5) to the Supplement.* |

*S5 Table, Operational definitions*

| *Term* | *Operational definition* |
| --- | --- |
| *Confounder* | *A common cause of the exposure and outcome of interest* |
| *Residual confounding* | *Persistent confounding after adjustment for known and measured confounders* |
| *Time-to-first event* | *The time between cohort entry and the event of interest* |
| *Recurrent event* | *Event that occurs more than once; in this study, recurrent events were defined as those occurring at least 14 days after the previous episode.* |

| *Reviewer #1 – Comments from the reviewer are included verbatim* | *Authors’ reply* |
| --- | --- |
| *“Regarding method section I didn’t find a data extraction tool with its description. I think it* *has to be incorporated in this section.”* | This study was conducted as part of a large population-based record-linkage study (with ~2 million children born July 2001-December 2012 in two states in Australia) that aimed to assess the effectiveness and impact of childhood immunisation program in Australia. Data extraction tools are used in systematic reviews so we do not understand the reviewer’s request. However, below we add some information that may answer the reviewer’s question.  The **data pipelines** for the linkage of databases are described narratively by co-authors Gidding (2017) and Moore (2016). Their papers were cited in the original submission. While a detailed description of the linkage processes is beyond the scope of this manuscript, these were summarised in the original submission. The revised manuscript includes (lines 94 to 104):  *We conducted a pre-registered (protocol DOI: 10.1371/journal.pone.0260388),^16^ retrospective, population-based record-linkage cohort study to examine the association between the type of first pertussis vaccine dose received in infancy and hospitalizations with a primary diagnosis coded as asthma (as per the tenth edition of the International Classification of Diseases [ICD-10 AM] coding scheme) for children aged between 5 to <15 years old. Details of the full cohort, datasets, data cleaning, and linkage procedures have been described elsewhere.^17,18^ Birth records were probabilistically linked to health data including immunization register and hospitalization data.17,18 The cohort was identified through the birth registries of the Australian states of New South Wales (NSW) and Western Australia (WA), and the NSW Perinatal Data Collection and WA Midwives’ Notification System. The linkage accuracy between birth register and immunization datasets was 99.0%, and between the birth registry and death data 96.6%.^17^*  A **data dictionary** was also included in the original submission (S4 Table). Variables presented in S1 Figure were mapped against the variables available in our dataset and further described. |
| *“Again, you fail to describe how to control data quality so you have to write it.”* | The Study Setting and Participants section has been edited accordingly and now reads (lines 103 to 104): *The linkage accuracy between birth register and immunization datasets was 99.0%, and between the birth registry and death data 96.6%.^17^* |
| *“In the data analysis and process section: some indispensable points are overlooked or not written like model fitness, what were you assumption to take candidate variables from bivariate to multivariate Cox regression statistical model, VIF, AND proportional hazard assumptions?”* | The methodology for the selection of covariates was governed by pre-specified causal assumptions presented in the protocol as DAGs. This is the standard of practice in modern epidemiology. The rationale was extensively discussed in our study protocol, as described in the section *Confounders and explanatory variables*. These notions are now reinforced in the revised version of this manuscript (lines 175-181): *Confounders and prognostically important covariates were selected a priori based on causal considerations, a prespecified DAG, and the modified disjunctive cause criterion.^16,24^ Statistical models were stratified by state of birth, allowing for different baseline hazards, and adjusted for socioeconomic status,^25^ remoteness, year and season of birth, birth order, maternal smoking during pregnancy, method of delivery, gestational age at delivery, Aboriginal status, and sex, as recorded on the birth registries and perinatal data collections, and measured before the administration of the first dose of wP/aP.* |
| *“Result section” “How much data you enrolled from that how much data you analyzed?”* | The cohort flow chart detailing the cohort ascertainment and exclusions was attached in the original submission (Figure 1) and included below  The analysis population was detailed in the original submission. It can be found in the following Tables:   1. Table 2: Hazard ratios and 95% confidence intervals for hospitalizations for asthma – Second column from left to right and footnote a. 2. Table 3: Hazard ratios and 95% confidence intervals for emergency department presentations for asthma in children born in New South Wales between 1997 and 1999 - Second column from left to right and footnote a. 3. Table 4: Hazard ratios and 95% confidence intervals for emergency department presentations for asthma in children born in Western Australia between 1997 and 1999 - Second column from left to right and footnote a. |

| *Reviewer #1 – Comments from the reviewer are included verbatim* | *Authors’ reply* |
| --- | --- |
| *“There are two tables that existed in your document but it doesn’t show the succession which means you put table 1 and table 1 so at least you need to show the continuity of the table by using the word “table 1 continued”.* | The reviewer is correct. We will liaise with the journal’s copy-editing team to correct this minor formatting issue in the final submission. |
| *“Since your study is about survival, where are the important graphs that show your study like* *Kaplan Meir survival estimate, cox regression table, and Comparison of survivorship functions for different categorical variables?”* | Kaplan Meir plots are included as Fig 2, Fig 3, and Fig 4, and supplementary S2 Figure. We designed this study based on the hypothesised causal mechanisms underpinning the effect of wP on the development of childhood asthma. The prespecified analyses were based on causal models, which were included in the study protocol. Therefore, the outputs presented in Tables 2, 3, and 4 include the “primary effect” of the exposure of interest on the outcome variable. **Causal estimands/survival functions for other variables, including confounders, were not under consideration and therefore beyond the scope of this analysis.** Additional information supporting our approach (i.e., avoiding the so-called Table 2 fallacy) can be found in Westreich & Greenland (2013): PMID: 23371353. |
| *“Discussion section” “It is shallow to reveal the detail of your findings”* | Additional edits have been now included (lines 310 to 317) to improve the discussion substantially. *In our study, treatment effects were estimated using HRs among children receiving wP as a first dose versus aP as a first dose, at least one dose of wP versus aP-only doses, and wP-only primary doses versus aP-only primary doses. A known limitation of the Cox model for the effect of medical interventions on failure time, is that hazards and HRs are not (and are not expected to be) constant over time. For these analyses and the causal question of interest, the HRs of our Cox model can only be interpreted as a weighted average of the true hazard ratios over the entire follow-up period.^32^ Alternative statistical approaches such as computing the restricted mean survival time in lieu of HRs or bootstrapping 95% CI are beyond the scope of this analysis and could be explored by future studies.^32^* |
| *References*  *Of the total of 3,4,13,14,15,20, and 22 references are outdated which is not appropriate.* | Here we provide the justification for each one of the references listed by the reviewer. Please note that the number of these references changed in the revised version of this manuscript.  Reference 3 (ref 8 in the revised version). Torvaldsen S, Hull BP, McIntyre PB. Using the Australian Childhood Immunisation Register to track the transition from whole-cell to acellular pertussis vaccines. Commun Dis Intell Q Rep. 2002;26(4):581–3. ***This paper describes the transition from whole-cell to acellular pertussis vaccination strategy in Australia. The transition process occurred between 1997 and 1999. This paper was published in 2002 and no further epidemiological descriptions have addressed the same topic. The paper remains relevant to our research (i.e., a population-based cohort study of Australian children born during the transition from wP to aP).***  Reference 4 (ref 9 in the revised version). McIntyre PB, Forrest J, Burgess M, Harvey B. Pertussis vaccines: past, present and future in Australia. Commun Intell. 1998;9(22):125–32. ***This paper was published in 1998, amidst the transition from wP to aP in Australia. It describes the implications of the introduction of aP in the Australian immunisation schedule and remains relevant to our research (i.e., a population-based cohort study of Australian children born during the transition from wP to aP).***  Reference 13 (ref 20 in the revised version). Cox DR. Regression models and life‐tables. J R Stat Soc Series B Stat Methodol. 1972;34(2):187–202. ***Here the Cox model was introduced for the first time to the scientific community. This paper is among the most-cited statistics papers ever and remains relevant to our research.***  Reference 14. Andersen PK, Gill RD. Cox’s regression model for counting processes: A large sample study. Ann Stat. 1982; 10(4):1100–20. ***Seminal paper from Andersen & Gill. Here, their model was introduced for the first time to the scientific community. This paper remains relevant to our research.***  Reference 15 (ref 21 in the revised version). Pink B. An Introduction to Socio-Economic Indexes for Areas (SEIFA) [Internet]. Canberra, ACT: Australian Bureau of Statistics; 2008 [cited 2021 Aug 6]. Available from: <https://www>. Ausstats.abs.gov.au/ausstats/subscriber.nsf/ **SEIFA is a suite of four summary measures that have been created from 2006 Census information. *This technical paper describes the SEIFA indexes, therefore it is relevant to our research.***  Reference 20 (ref 33 in the revised version). Mascart F, Hainaut M, Peltier A, Verscheure V, Levy J, Locht C. Modulation of the infant immune responses by the first pertussis vaccine administrations. Vaccine. 2006 Jan;25(2):391–8. doi: 10.1016/j.vaccine.2006.06.046. ***To date, two clinical studies (Mascart et al, 2006 & Holt et al, 2016) provide evidence of bystander stimulation of immunity against food allergens among aP versus wP vaccinees. These studies underpin the rationale of our research. Therefore, citing this work is appropriate.***  Reference 22 (ref 35 in the revised version). Nilsson L, Kjellman NI, Bjorksten B. Allergic disease at the age of 7 years after pertussis vaccination in infancy: Results from the follow-up of a randomized controlled trial of 3 vaccines. Arch Pediatr Adolesc Med. 2003 Dec;157(12):1184–9. doi: 10.1001/archpedi.157.12.1184. ***This paper reports the 7-year follow-up data of the only randomised controlled trial comparing the effect of wP versus aP on atopic outcomes. This paper remains relevant to our research.*** |

**REVIEWER # 2**

| *Reviewer #2 – Comments from the reviewer are included verbatim* | *Authors’ reply* |
| --- | --- |
| *“In the present study, the authors evaluated hypothesis that whole-cell pertussis (wP) vaccination in early infancy might protect against atopic asthma in childhood.*  *The manuscript is interesting and, in general, fair written.”* | We thank the reviewer for their time and comments. We are glad that they found our manuscript interesting and welcome their feedback. |
| “1. The background section introduced some relevant articles. Please explain the results or summarize with effect sizes.” | The cited immunological study assessed T cell and antibody responses to *B. pertussis* antigens and defined epitope repertoires using PBMCs from volunteers originally primed with either the wP or aP vaccine (Bancroft et al, 2016). Polarization of T cell responses was measured by the ratio of IL-5/IFNγ upon stimulation with epitope pools. The study found that the dominant cytokine secreted was IFN-γ in wP donors, (median 5, IQR 2 to 31) while in aP donors, responses were biased towards IL-5 production (median 0.4, IQR 0.1 to 2.5) (Bancroft et al, 2016). Since we are reporting a population-based/epidemiological study, we feel that adding the medians and IQRs of IL-5/IFNγ may not be intuitive for readers of this paper.  For the case-control study of Estcourt et al, we reported the adjusted OR (A case-control study of children born during this period, found that those with allergist-diagnosed IgE-mediated food allergy were less likely than matched controls to have received wP vaccine as their first dose (odds ratio 0.77; 95% confidence interval [CI] 0.62–0.95).(12) The findings raised the question of whether a similar association might also be observable for atopic asthma.”) |
| “2. Authors used ICD-10 AM diagnostic codes. I wonder do they also have old ICD-9 codings or if all of the data were originally set to ICD-10.” | The hospitalization included in this cohort study were all coded with ICD-10 codes. This is because the transition from ICD-9 CM to ICD-10 AM occurred in a stepwise fashion, which commenced in NSW, the Australian Capital Territory, Victoria, and the Northern Territory from July 1998, and the remaining Australian jurisdictions from July 1999. Therefore, by the time of cohort entry (i.e., between 1 Jan 2002 and 31 Dec 2004) ICD-9 CM was fully replaced by the implementation of ICD-10 AM in NSW and WA. Lines 147 to 151 of the revised manuscript now read: *For NSW-born children, asthma presentations to the emergency department were ascertained from the NSW Emergency Department Data Collection, with diagnoses coded using SNOMED-CT and ICD-10 AM [in lieu of ICD-9 AM] (S2 Table).* ICD-9 CM codes were also removed from the Supplement (S2 table) |
| “3. In the ‘Statistical Analysis’ section, I suggest authors to also refer open access articles than current closed access articles, for readers who are interested.” | We thank the reviewer for this comment. We now cite Terry Therneau’s vignette, which is available on CRAN as an open-access pdf, and Kleibaun and Klein’s survival analysis textbook, which can be found as an open-access pdf through the University of Peshawar. |
| “4. What is the future scope of the proposed research, authors have described the limitations in a good way, and I suggest that these can be the future scope of the work.” | We agree with the reviewer. We have now added a statement (lines 317 to 319) in the discussion highlighting the future scope of our work – ‘Alternative statistical approaches such as computing the restricted mean survival time in lieu of HRs or bootstrapping 95% CI are beyond the scope of this analysis and could be explored by future studies’. |
