# Supplementary material for: Association between pertussis vaccination in infancy and childhood asthma: A population-based record linkage cohort study
Source: PLoS One. 2023 Oct 4;18(10):e0291483. doi: 10.1371/journal.pone.0291483 (PMC10550153; doi:10.1371/journal.pone.0291483)
Supplement: Attachment — Submitted filename: Response to Reviewers.docx [file pone.0291483.s023.docx]

Response to the Editor’s comments

This is an excellent paper. Please see my minor comments.

- Response: We thank the Editor for their feedback and comments.

#1. Primary and secondary complete-case analyses (i.e., including only observations with no missing data) were performed to estimate unadjusted and adjusted hazard ratios (HRs), and their 95% CIs -> Please cite the statistical guideline (DOI: [https://doi.org/10.54724/lc.2023.e8](https://protect-au.mimecast.com/s/e-zhCwV1vMfv780kRfK36sL?domain=doi.org)).

- Response: The revised version of our manuscript includes the above-mentioned citation (i.e., ref 24).

#2. Please add this sentence. A two-sided P less than 0.05 is considered significant.

- Response: We value the suggestion from the Editor. Nevertheless, given the multiplicity of testing, we are refraining from designating a p-value of <0.05 as indicating statistical significance.

#3. Please add IRB registration number. This study was approved by the human research ethics committees of the Department of Health of WA, NSW Population Health Service, Australian Institute of Health and Welfare, Curtin University (Number XXX), the WA Aboriginal Health Ethics Committee (Number XXX), and the Aboriginal Health and Medical Research Council Ethics Committee (Number XXX). This is an excellent paper!!

- Response: We thank the Editor for their feedback. We have edited this section and included the approval numbers: This study was approved by the human research ethics committees of the Department of Health of WA, (approval number: 2012/75), NSW Population Health Service, (approval number: HREC/13/CIPHS/15), Australian Institute of Health and Welfare, (approval number: EC2012/4/62), Curtin University, (approval number: HRE2019-0350), the WA Aboriginal Health Ethics Committee, (approval number: 459), and the Aboriginal Health and Medical Research Council Ethics Committee (approval number: 931/13). A waiver of consent was requested and granted owing to the large size of the study cohort.
